# Superior Mesenteric Artery Syndrome due to a Vertebral Hemangioma and Postpartum Osteoporosis following Treatment

**DOI:** 10.1155/2015/930534

**Published:** 2015-01-05

**Authors:** Mehmet Elmadag, Yunus Güzel, Gokcer Uzer, İbrahim Tuncay

**Affiliations:** ^1^Department of Orthopaedics and Traumatology, School of Medicine, Bezmialem Vakif University, Adnan Menderes Boulevard, Vatan Street, Fatih, 34093 Istanbul, Turkey; ^2^Department of Orthopaedics and Traumatology, School of Medicine, Ordu University, Cumhuriyet Campus, 52200 Ordu, Turkey

## Abstract

In pregnancy, advanced vertebral hemangiomas may be seen, and these require treatment. The case reported here is of a 35-year-old female in the 32nd week of pregnancy who was admitted to the orthopaedics clinic with a history of backache and difficulty walking. A burst fracture of L1 associated with a vertebral hemangioma was identified with an L3 compression fracture secondary to osteoporosis. The local kyphosis angle between T12 and L2 was 27°. Kyphotic deformity was corrected and postoperatively, the measured T12–L2 local kyphotic angle was 9°. Twelve hours postoperatively, oral nutrition was allowed, but she developed nausea and vomiting and twenty-four hours postoperatively, an electrolyte imbalance developed. Postoperatively, the patient was diagnosed with superior mesenteric artery syndrome. To the best of our knowledge, this is the first reported case of superior mesenteric artery syndrome, which occurred following the correction of a kyphotic deformity that had developed secondary to an advanced hemangioma in pregnancy.

## 1. Introduction

Hemangiomas are one of the most common vertebral neoplasms and are usually asymptomatic. The rate of asymptomatic vertebral hemangiomas in the general population is approximately 10% [1, 2]. Hemangiomas may be spread throughout the spine but tend to be more symptomatic in the lower thoracic or lumbar spine. They cause pain that can be related to an osseous expansion or a pathological fracture and neurological deficits that are caused by compression of the neural elements [3]. In pregnancy, advanced hemangiomas may be seen and these require treatment [4]. Superior mesenteric artery (SMA) syndrome, first defined by Rokitansky [5] in 1842, occurs as a result of compression of the third section of the duodenum between the aorta and the SMA. This is a very rare complication which occurs following the correction of pediatric spinal deformities. It should be borne in mind that patients can experience rapid weight loss after surgery, if oral nutrition is not tolerated. To the best of our knowledge, this is the first reported case of SMA syndrome, which occurred following the correction of a kyphotic deformity that had developed secondary to an advanced hemangioma in pregnancy.

## 2. Case Report

A 35-year-old female in the 32nd week of pregnancy had presented at another center because of backache and difficulty walking. A burst fracture of L1 associated with a vertebral hemangioma was identified with an L3 compression fracture secondary to osteoporosis. Surgery and early birth were recommended, but the patient refused and left that center. Three months after delivery, she presented at our clinic as the complaints of back pain were increasing. The physical examination revealed an otherwise healthy individual with no history of disease before the pregnancy. She was 165 cm tall and weighed 63 kg, with a body mass index (BMI) of 23. It was understood from the patient history that, with weight loss after the birth, she had returned to her previous BMI. There was a Frankel D deficit in both lower extremities according to the Frankel classification, no ankle clonus and a negative Babinski reflex.

**Figure 1:**
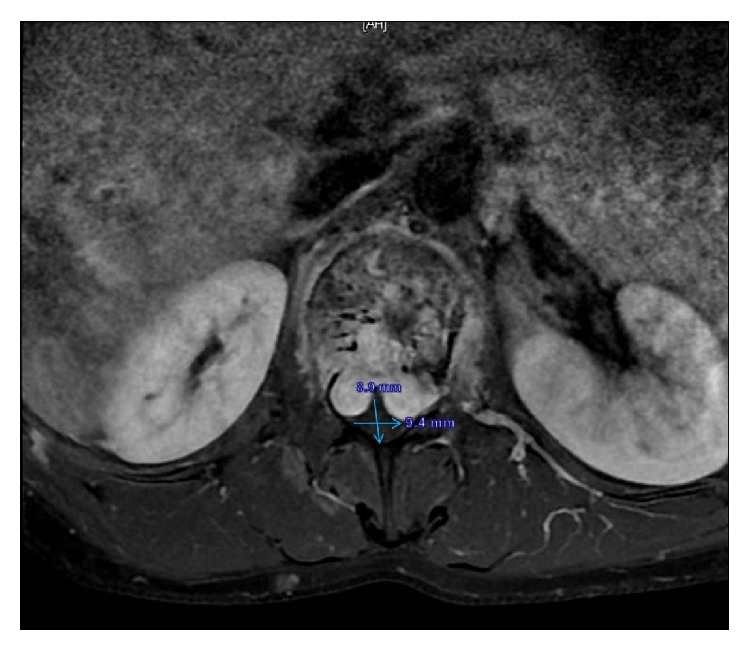
A burst fracture of L1 associated with a vertebral hemangioma causing canal compression with expansion of the L1 body.

**Figure 2:**
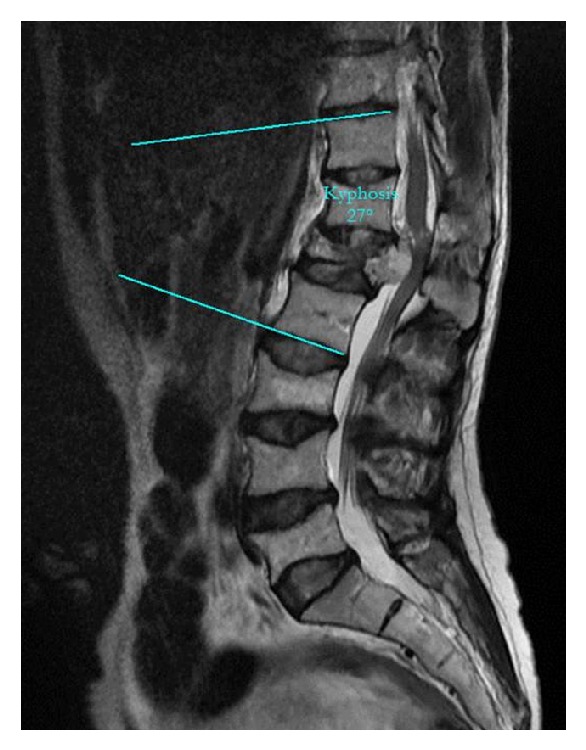
Preoperatively, the measured T12–L2 local kyphotic angle.

**Figure 3:**
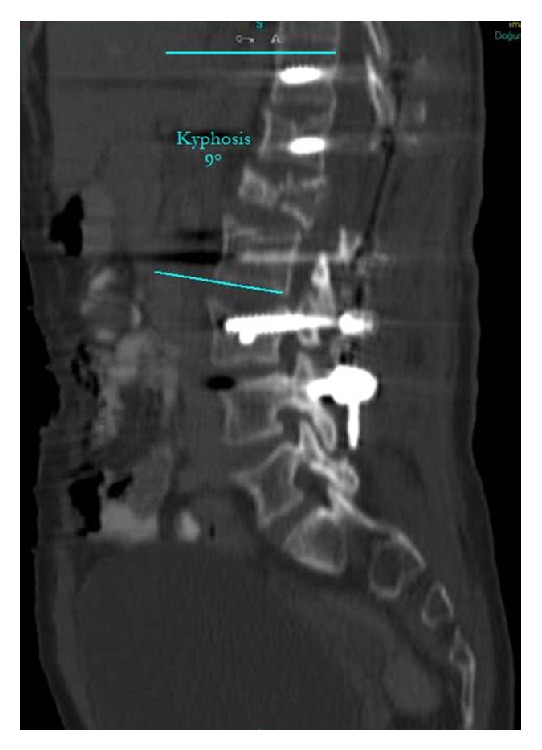
Postoperatively, the measured T12–L2 local kyphotic angle.

Pain increased on palpation of the back. The patient had difficulty standing and waking without support. Radiographs showed a burst fracture (the vertebral posterior complex was intact, but the fracture was in the anterior and middle compartment) of L1 associated with a vertebral hemangioma causing canal compression with expansion of the L1 body and a compression fracture of L3 secondary to postpartum osteoporosis (Figure 1). The *T* score was −3.5 SD on bone densitometry. The local kyphosis angle between T12 and L2 was 27° (Figure 2). Under general anesthesia, total decompression was performed with an L1 laminectomy and dural cut. During decompression, there was blood loss of approximately 500 mL, which was controlled by surgical packing at intervals. A total of 3 cc bone cement was injected with a vertebroplasty device (Kyphon, Sunnyvale, CA) to shrink the hemangioma of the L1 vertebra. The vertebra was stabilized with instrumentation with T11–L4 posterior pedicle screws without placing a screw in the L1 pedicle. Using the same system, the acute kyphotic deformity was corrected. Postoperatively, the measured T12–L2 local kyphotic angle was 9° (Figure 3). Twelve hours postoperatively, oral nutrition was allowed, but she developed nausea and vomiting. The oral nutrition was stopped and supportive treatment was started with intravenous serum.

**Figure 4:**
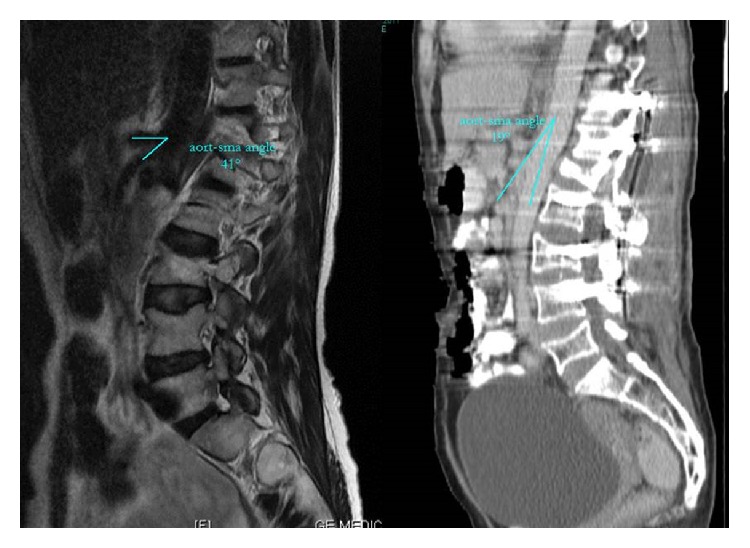
Preoperative and postoperative angle of superior mesenteric artery in computerized tomography.

Twenty-four hours postoperatively, an electrolyte imbalance developed and general surgery and gastroenterology consultations were requested. As the patient's general condition was poor, she was not mobilized with a thoracolumbar stabilization orthosis to allow mobilization. Blood pressure, pulse, temperature, electrolytes, and liver enzymes were monitored and serum was administered to restore the electrolyte balance. An endoscopic examination showed no ulcer, gastritis, or stenosis. On postoperative day 3, a psychiatric consultation evaluated her as normal, and the gastroenterology consultation was repeated. From the preoperative computed tomography (CT) images and postoperative contrast abdominal CT, it was determined that the angle of the SMA to the aorta had decreased from 41° to 29° (Figure 4) and the third section of the duodenum had undergone stenosis in the area of this bifurcation.

Postoperatively, she was diagnosed with SMA syndrome. For 7 days, oral intake was halted and TPN was continued. The electrolytes were monitored daily. The blood urea nitrogen (65 mg/dL) and creatinine (1.8 mg/dL), which were high in the early postoperative period, started to decrease on postoperative day 7 and oral nutrition with liquid food was then allowed. The patient was discharged on postoperative day 10. At the 12-month follow-up, her general condition was good and the blood parameters were normal. With minimal back pain, there was no change in the early postoperative sagittal balance at the 12-month follow-up.

## 3. Discussion

Virchow first defined a vertebral hemangioma [6]. Hammes and Junghanns [1, 2] reported that 10% of the population have asymptomatic vertebral hemangiomas. Balado reported an advanced vertebra hemangioma in pregnancy in 1927 [7]. Progression is generally seen during the third trimester of pregnancy. Myelopathic symptoms can develop in these cases, and treatment can involve radiotherapy, embolization, vertebroplasty, and combined surgery [8–11].

The hemodynamic and hormonal changes in pregnancy cause progression of the hemangioma. The increased circulatory volume in pregnancy and activation of estrogen and progesterone receptors in the hemangioma cause growth of the hemangioma. These changes occur mostly during the third trimester.

The SMA generally originates from the aorta at the level of the first lumbar vertebra, forming an exit angle at a mean of 35–60°. There is a space of 13–34 mm between the SMA and the aorta, and the third section of the duodenum crosses here. When the aortomesenteric angle decreases, the duodenum may be narrowed.

Some events can reduce the aortomesenteric angle, such as lumbar hyperlordosis and extended spine positioning and vertebra deformity following surgery. This occurs mostly in tall, thin subjects, rather than obese patients. It is frequent in patients who have undergone marked weight loss over a short period of time, in those with prolonged inadequate nutrition, and in those with reduced retroperitoneal fat.

Another reason for duodenal obstruction is the surgical treatment of spinal deformities. Correction of an acute spinal curvature and extension of body length can lead to compression of the duodenum by reducing the angle between the SMA and the aorta. In literature over the last 50 years, this situation has almost always been reported to develop secondary to surgery for vertebra deformities, such as scoliosis and kyphosis, and is seen in adolescents. The case presented here differs in that surgical treatment was applied to a kyphotic deformity, which developed due to a vertebra fracture secondary to postpartum osteoporosis and growth of a vertebral hemangioma during pregnancy in a slim, young, adult female. The diagnosis resulted from complaints that started on the first postoperative day. Therefore, in many ways, this case is unique.

The most frequent symptoms of SMA syndrome are nausea, vomiting, and loss of appetite, which start during the early postoperative period. These complaints can often last for weeks and be associated with loss of weight. Indigestion and abdominal distension can occur. Partial obstruction of the duodenum, causing vomiting, distension, and duodenal edema, can progress to full obstruction. The electrolyte imbalance results in oliguria and death might result from hemodynamic shock. The patient presented here developed high blood urea nitrogen and creatinine levels.

In patients with a confirmed diagnosis, after decompression with a nasogastric tube and administration of antispasmodics, oral nutrition is stopped and parenteral nutrition is started. After passing through the acute phase and correcting the electrolyte imbalance, oral nutrition is allowed and after determining the most suitable position for eating, that position should be adopted [12, 13]. The retroperitoneal fat tissue starts to reach a suitable thickness with oral intake, and nitrogen balance is obtained. After the addition of solid foods, the weakened abdominal muscles become strengthened and the lumbar lordosis returns to normal. In the case presented here, parenteral nutrition was used until the patient's general condition and electrolyte imbalance were corrected.

In conclusion, although SMA syndrome, which can develop in the early period following correction of kyphotic pathologies of the vertebrae, is more often seen in adolescents, the clinician should remember that it can also be seen in adults.
